# Comparison of feasibility and efficacy of microsurgery and interventional techniques in the management of unruptured middle cerebral artery aneurysms with a history of transient ischemic attack

**DOI:** 10.3389/fsurg.2026.1832695

**Published:** 2026-05-11

**Authors:** Te Li, Zhong Lin Li, Yin Ren, Manyi Xie

**Affiliations:** Department of Neurosurgery, The Affiliated Hospital of Xuzhou Medical University, Xuzhou, Jiangsu, China

**Keywords:** complete occlusion, complications, recurrence, transient ischemic attack, unruptured middle cerebral artery aneurysm

## Abstract

**Objective:**

To compare the feasibility and efficacy of microsurgery and interventional techniques in the treatment of unruptured middle cerebral artery aneurysms (MCAA) with a history of transient ischemic attack (TIA).

**Methods:**

A prospective cohort comparative study was conducted using 100 patients with unruptured MCAA and a history of TIA who were admitted to our hospital from January 2023 to October 2024. The patients were divided into an interventional group and a microsurgical group, with 50 patients in each group. The interventional group underwent endovascular embolization, while the microsurgical group received microsurgical treatment. Various surgical outcomes were compared between the two groups, including procedure time, blood loss, postoperative hospital stay, and treatment costs. Rates of complete and incomplete aneurysm occlusion, treatment-related serious adverse events within 30 days after surgery, functional outcomes at 6 months after surgery as measured by the modified Rankin Scale (mRS), levels of miR-27a and miR-143, and recurrence rates at 1 year after surgery were also analyzed. Additionally, the cure rates for aneurysms in terms of shape and location were compared between the two groups.

**Results:**

In the interventional group, the operation time, blood loss, and postoperative hospital stay were lower than those in the microsurgical group. However, the treatment cost was higher in the interventional group (*P* < 0.05). The complete occlusion rate in the interventional group was 88.00%, which was lower than 100.00% in the microsurgical group (*P* < 0.05). The incidence of treatment-related SAEs within 30 days after surgery was 4.00% in the interventional group, compared to 16.00% in the microsurgical group (*P* < 0.05). At 6 months postoperatively, the rate of good functional outcomes was 92.00% in the interventional group, similar to 98.00% in the microsurgical group, with no statistically significant difference between the two groups (*P* > 0.05). Compared to preoperative levels, both groups showed significantly increased miR-27a and miR-143 levels at 3 days postoperatively (*P* < 0.05). However, the levels of miR-27a and miR-143 were lower in the interventional group at 3 days postoperatively than in the microsurgical group (*P* < 0.05). The recurrence rate at 1 year was 12.00% in the interventional group, compared to 0% in the microsurgical group (*P* < 0.05). For aneurysms with complex anatomies (wide neck, apex-neck ratio < 1.5, involvement of branches, or irregular shape), the complete occlusion rate after interventional treatment was significantly lower, and the 1-year recurrence rate was significantly higher (*P* < 0.05).

**Conclusion:**

Both microsurgery and interventional techniques are feasible therapeutic strategies for patients with unruptured MCAA and a history of TIA. Interventional techniques have the advantages of shorter operation time, less blood loss, faster postoperative recovery and higher perioperative safety, whereas microsurgery yields a higher complete occlusion rate and lower recurrence risk.

## 1 Introduction

Unruptured middle cerebral artery aneurysms (MCAA) are common clinical cerebrovascular diseases with a potential risk of rupture, which can lead to subarachnoid hemorrhage and even life-threatening consequences once ruptured (1, 2). For patients with a history of transient ischemic attack (TIA), their cerebrovascular environment may be complicated by atherosclerosis and vascular endothelial dysfunction, making the management of their aneurysms more complex and the clinical decision-making more challenging (3, 4). Microsurgical clipping is a traditional clinical intervention that directly occludes the aneurysm neck via craniotomy, achieving a favorable cure rate; however, this invasive procedure is associated with a relatively high risk of complications (5, 6). Endovascular interventional therapy, a minimally invasive technology widely promoted in clinical practice in recent years, embolizes aneurysms by placing stents and coils through catheters, which has the advantages of minimal invasion and rapid recovery. Nevertheless, clinical practice has found that this minimally invasive technique is prone to incomplete occlusion and a relatively high risk of long-term recurrence (7, 8). Current studies have confirmed that both microsurgical clipping and endovascular interventional embolization achieve good therapeutic effects in the treatment of MCAA. Although endovascular interventional therapy is less invasive, it has a higher recurrence rate (9, 10). However, there is no consensus on the optimal therapeutic regimen for the specific population of patients with a history of TIA, which requires a comprehensive trade-off between the cure rate of microsurgery, the safety of endovascular interventional therapy and their respective impacts on prognosis. Studies have reported that microRNAs are closely associated with the occurrence of brain injury, and the detection of their expression changes can predict the prognosis of patients with aneurysmal subarachnoid hemorrhage (11). Therefore, this study aimed to systematically compare the feasibility and efficacy of microsurgery and endovascular interventional embolization in the management of unruptured MCAA with a history of TIA, and provide molecular evidence by analyzing biochemical indicators such as microRNAs, so as to offer a reference for the formulation of therapeutic strategies for this high-risk population.

## 2 Materials and methods

### 2.1 Study subjects

The sample size was determined based on previous research experience and statistical analysis: previous studies have shown that both endovascular interventional therapy and microsurgical clipping achieve good efficacy in patients with unruptured MCAA and a history of TIA, with complete occlusion rates of 76.30% and 90.40% respectively at the last follow-up (12). This study intended to compare the efficacy of endovascular interventional therapy and microsurgical clipping in the treatment of unruptured MCAA with a history of TIA, with the complete occlusion rate as the primary outcome measure for sample size estimation. With a significance level (*α*) of 0.05 and a test power of 0.9, and the preset complete occlusion rates of the two groups as 0.75 and 0.9 respectively, statistical software calculated the required sample size as 92 cases. Considering a potential loss to follow-up rate of approximately 10%, a total of 100 patients with unruptured MCAA and a history of TIA admitted to our hospital from January 2023 to October 2024 were enrolled in this prospective study.

Inclusion criteria: ① Conforming to the diagnostic criteria for unruptured intracranial aneurysms (13), with a single unruptured MCAA at the M1 or M2 segment of the middle cerebral artery confirmed by digital subtraction angiography (DSA); ② A confirmed history of one or more TIA episodes within 6 months before admission; ③ Aged 18 to 75 years; ④ Tolerable to general anesthesia and microsurgical clipping or endovascular interventional surgery; ⑤ Preoperative mRS score ≤ 2 points.

Exclusion criteria: ① TIA caused by non-atherosclerotic factors such as atrial fibrillation, valvular heart disease and vasculitis; ② Complicated with other intracranial aneurysms or arteriovenous malformations requiring simultaneous treatment; ③ Fusiform, dissecting or infectious aneurysms; ④ A history of ipsilateral craniotomy or endovascular interventional therapy; ⑤ Accompanied by massive hematoma requiring immediate decompression; ⑥ Pregnant or lactating women; ⑦ Suffering from severe mental illness or with an expected survival time of less than 1 year; ⑧ Complicated with severe hepatic, renal, cardiac, pulmonary dysfunction or coagulation disorders; ⑨ Lost to follow-up.

Initially, this study planned to use a computer-generated random number table for simple random grouping. However, in practice, it was not possible to implement a strict allocation concealment mechanism, nor could the surgeons and patients be blinded to their assigned groups. Additionally, some patients could not accept the randomly assigned treatment due to the anatomical characteristics of their aneurysms (e.g., wide-neck aneurysms) or personal preferences regarding treatment. As a result, the study design was adjusted to a prospective cohort comparison study. Patients in both groups were assigned treatments based on the comprehensive evaluation by clinicians and the patients' own choices. The research team ensured comparable baseline characteristics between the two groups by matching them based on demographic data, aneurysm anatomy, and comorbidities. Ultimately, 50 patients were included in each group.

The patients were randomly divided into the interventional group and the microsurgical group with 50 cases in each group using a computer-generated random number table. There were no statistically significant differences in baseline data between the two groups (*P* > 0.05), indicating good comparability (Table 1). This study was approved by the Medical Ethics Committee of our hospital, and all patients and their family members provided informed consent and signed written informed consent forms.

**Table 1:** Comparison of general clinical data between the two groups [*n*(%), $\left(\bar{x}\pm s\right)$].

Group	Number of examples	Gender	Age (years)	Time from last TIA to hospital admission (d)	Aneurysm diameter (mm)		Aneurysm location		
		Male	Female				M1	M2	Bifurcation
Intervention Group	50	32 (64.00%)	18 (36.00%)	64.90 ± 5.06	46.55 ± 10.80	5.20 ± 0.32	15 (30.00%)	6 (12.00%)	29 (58.00%)
Microarray	50	35 (70.00%)	15 (30.00%)	65.81 ± 4.70	48.90 ± 12.15	5.30 ± 0.40	13 (26.00%)	5 (10.00%)	32 (64.00%)
*χ*²/t	–	0.407	0.932	1.022	1.380	0.186			
P	–	0.523	0.354	0.309	0.171	0.839			

### 2.2 Therapeutic methods

All patients underwent cranial computed tomography angiography (CTA) or DSA after admission to determine the size, orientation of the aneurysm and the degree of cerebral vasospasm, and surgical risk assessment was performed. Patients without surgical contraindications underwent surgery within 72 h after admission. Routine comprehensive postoperative treatments were administered to both groups, including dehydration and intracranial pressure reduction (e.g., mannitol), prophylactic anti-infection, and prevention and treatment of cerebral vasospasm (e.g., nimodipine).

#### 2.2.1 Interventional group

**Figure 1:**
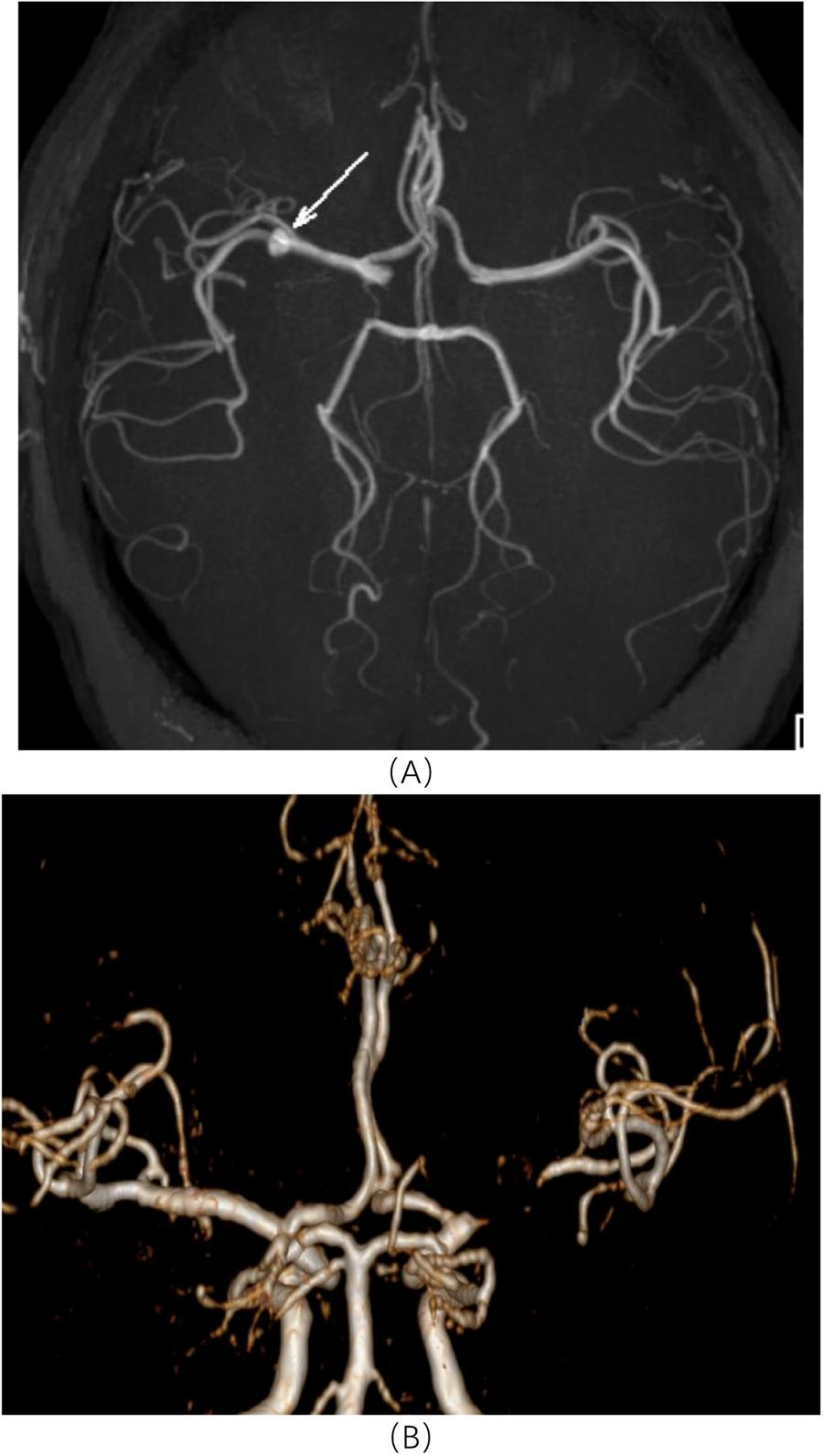
Radiographic image of a patient treated using endovascular embolization (Male patient, 67 years old). **(A)** represents preoperative head DSA images, and **(B)** represents postoperative 3D head DSA images.

Endovascular interventional embolization was performed. In this study, patients in the intervention group were all treated with simple coiling alone. No modern neurointerventional techniques such as stent-assisted embolization, balloon angioplasty, or flow-diverting devices were used. Aneurysm occlusion was achieved in all cases through simple coiling.The patients received a loading dose of aspirin enteric-coated tablets (300 mg) plus clopidogrel (300 mg) orally 4 h before surgery. Heparin was intravenously injected at the start of surgery (routinely 60–100 IU/kg), followed by continuous intravenous pumping of a maintenance dose to maintain the activated clotting time at 250–300 s throughout the operation. General intravenous anesthesia was administered. Percutaneous femoral artery puncture was performed using the modified Seldinger technique, and a 7.5 F arterial sheath was successfully placed. Under the guidance of a guide wire, a Tracker catheter was super-selected to the affected internal carotid artery for DSA to evaluate the morphology, size, neck width of the aneurysm and its relationship with the parent artery. The size of the aneurysm was measured to select matching coils with appropriate diameter and length. Under roadmap guidance, the tip of the microcatheter was placed at an ideal position in the aneurysm sac. Coils were sequentially delivered into the aneurysm sac for dense packing until DSA showed no contrast medium filling in the aneurysm sac, achieving Raymond grade I occlusion. After embolization, protamine and heparin were intravenously injected at a dose ratio of 1:1. The catheters and sheath were withdrawn, and the puncture site was compressed and bandaged. A typical case is shown in Figure 1.

#### 2.2.2 Microsurgical group

**Figure 2:**
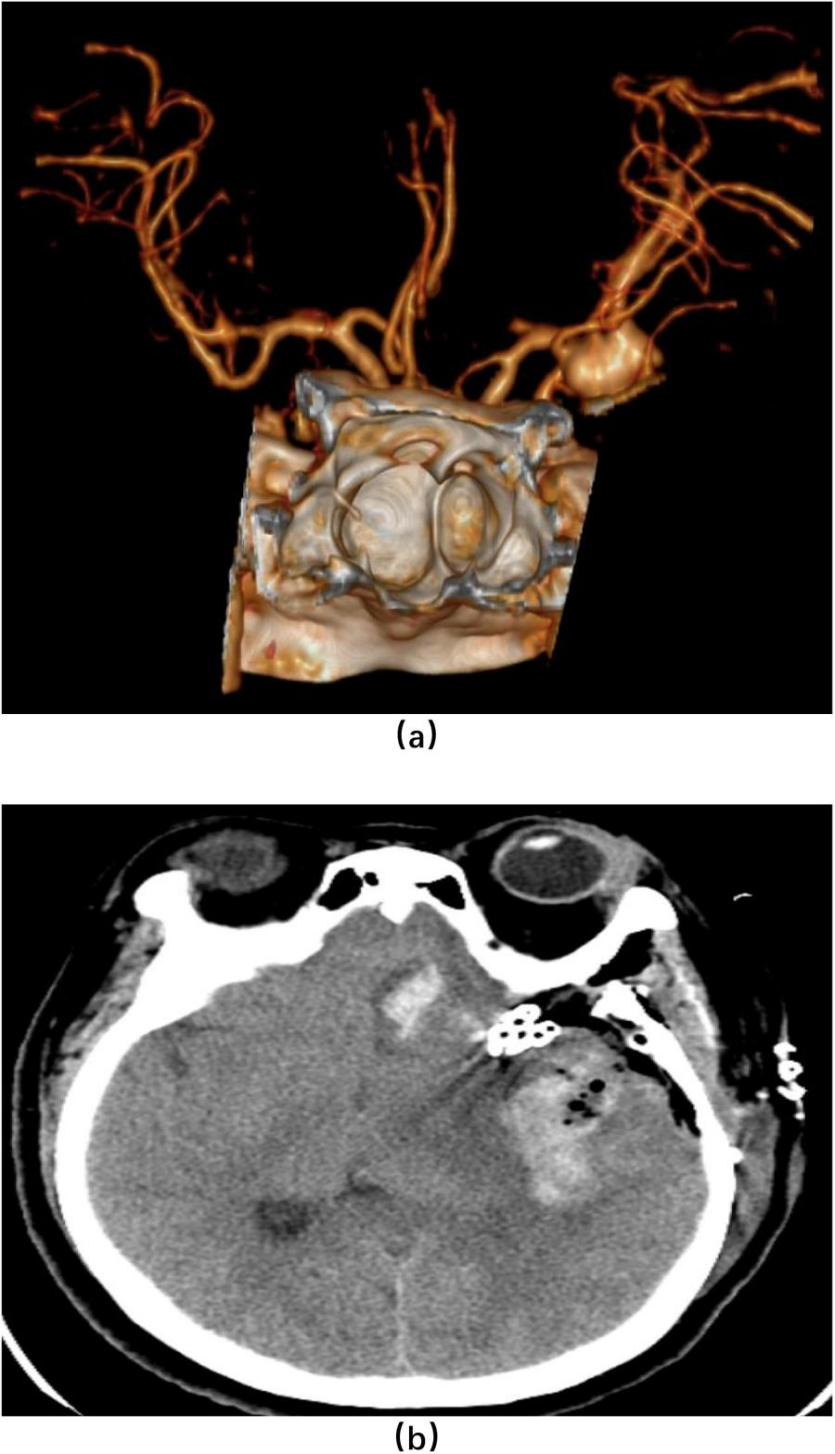
Imaging of a patient treated with microsurgical clipping (male, 65 years old). **(a)** shows preoperative head DSA; **(b)** shows postoperative three-dimensional CT of the head.

Microsurgical clipping was performed. The patients were placed in the supine position and received general anesthesia with tracheal intubation. Standard pterional craniotomy was adopted. The skin and soft tissues were incised layer by layer, and the skin flap was bluntly dissected. A burr hole was drilled in front of the junction of the coronal suture and the superior temporal line, a bone flap was formed with a milling cutter, and approximately two-thirds of the sphenoid ridge was resected with a burr to expose the skull base. The dura mater was incised in an arc shape. Cerebrospinal fluid was slowly released under a microscope, the sylvian fissure was sharply dissected, and the frontal and temporal lobes were retracted to reduce brain tissue tension. Dissection was performed distally along the internal carotid artery to locate the middle cerebral artery and the aneurysm. The arachnoid around the aneurysm neck was sharply dissected to fully expose the aneurysm neck. Temporary clips were used to occlude the proximal and distal segments of the parent artery as needed. According to the neck width and morphology of the aneurysm, an appropriately sized permanent aneurysm clip was selected to occlude the aneurysm neck under direct vision. If the clipping position was unsatisfactory or there was residual aneurysm, adjustments were made; for wide-necked aneurysms, reconstructive clipping with multiple aneurysm clips was performed. The temporary clips were released, and the patency of the parent artery and its branches as well as the complete occlusion of the aneurysm were confirmed by intraoperative fluorescent angiography or microvascular ultrasound. After strict hemostasis, the dura mater was watertightly sutured. If there was no obvious brain tissue swelling after surgery, the bone flap was repositioned and fixed; if there was obvious brain herniation, decompressive craniectomy was performed. Finally, the scalp was sutured layer by layer and covered with sterile dressings. A typical case is shown in Figure 2.

### 2.3 Outcome measures

#### 2.3.1 Perioperative indicators

Operation time, blood loss, postoperative hospital stay and treatment cost were recorded.

#### 2.3.2 Aneurysm occlusion rate

Aneurysm occlusion was evaluated by DSA immediately after surgery: complete occlusion was defined as no contrast medium filling in the aneurysm sac (Raymond grade I) or only a minimal amount of contrast medium residue at the root of the aneurysm neck (Raymond grade II) (14); incomplete occlusion was defined as any contrast medium filling in the aneurysm sac (Raymond grade III).

#### 2.3.3 Treatment-related serious adverse events (SAEs) within 30 days after surgery

SAEs included intracranial infection, epileptic seizures, myocardial infarction, ischemic and hemorrhagic stroke. Ischemic stroke was defined as the occurrence of new neurological deficits within 30 days after surgery, with new infarct foci confirmed by diffusion-weighted imaging (DWI) of cranial MRI or CT. Hemorrhagic stroke was defined as the occurrence of new intracranial hemorrhage (including parenchymal hematoma, subarachnoid hemorrhage, etc.) confirmed by postoperative cranial CT or MRI, regardless of the presence of clinical symptoms.

#### 2.3.4 Good functional prognosis rate at 6 months postoperatively

The mRS score was assessed by outpatient or telephone follow-up at 6 months after surgery to evaluate the prognosis of patients (15). The mRS scoring criteria were as follows: 1 point = symptomatic but no obvious disability; 2 points = mild disability, unable to resume pre-illness activities but able to handle personal affairs without assistance; 3 points = moderate disability, requiring some assistance in daily life but able to walk without assistance; 4 points = severe disability, unable to take care of oneself in daily life and functions; 5 points = profound disability, unable to take care of oneself in daily life and functions with incontinence; 6 points = death. A mRS score ≤ 2 points was defined as a good functional prognosis.

#### 2.3.5 Serum levels of miR-27a and miR-143

Fasting peripheral venous blood samples (3 mL) were collected from all patients within 24 h before surgery and at 3 days after surgery, and placed in EDTA-anticoagulant blood collection tubes. The samples were centrifuged at 2500 r/min for 5 min, and the serum was extracted and centrifuged again to remove precipitates and lipid layers. A total of 250 μL of serum was added to SBI precipitant, and the precipitate was obtained by centrifugation, mixed with 50 μL of PBS buffer and vortexed to form exosome suspension. Lysis buffer (700 μL) was added to the exosome suspension and mixed well, followed by incubation at room temperature (about 25 ℃) for 5 min; trichloromethane (140 μL) was added and incubated for another 2 min; then the mixture was centrifuged at 4 ℃ for 15 min, and 350 μL of the upper aqueous phase was transferred to a sterile tube, mixed with 675 μL of anhydrous ethanol, added with eluent, and eluted repeatedly for 3 times to obtain RNA. The RNA concentration was determined by a Nano Drop spectrophotometer. Total RNA was separated and purified by gel electrophoresis and then reverse-transcribed into cDNA. Using cDNA as a template, the expression levels of miR-27a and miR-143 were detected by a miRNA fluorescence quantitative kit. With *β*-actin as the internal reference, the relative expression levels were calculated by the 2-*ΔΔ*Ct method, and the experiment was repeated 3 times to take the average value.

#### 2.3.6 Recurrence rate

Cranial CTA was rechecked at 1 year after surgery. Recurrence was defined as the appearance of any new contrast medium filling in the previously occluded aneurysm sac (i.e., coil compaction or aneurysm recanalization) or the reoccurrence of aneurysmal shadow at or distal to the aneurysm clip site in the follow-up imaging compared with the immediate postoperative angiography.

### 2.4 Statistical analysis

All data were processed using the SPSS 25.0 statistical software. The Shapiro–Wilk test was used to test the normality of continuous variables. Normally distributed measurement data were expressed as (x ± s), and intergroup comparisons were performed using the independent samples *t*-test. Count data were expressed as rates (%), and intergroup comparisons were performed using the *χ*² test; the Fisher's exact probability test was used when the theoretical frequency of any cell in the 4 × 4 contingency table was <5. All hypothesis tests were two-tailed, with a significance level of *α* = 0.05. A *P* value < 0.05 indicated a statistically significant difference.

## 3 Results

### 3.1 Comparison of perioperative indicators between the two groups

The operation time, blood loss and postoperative hospital stay in the interventional group were significantly lower than those in the microsurgical group, while the treatment cost was significantly higher (all *P* < 0.001, Table 2).

**Table 2:** Comparison of perioperative indicators between the two groups $\left(\bar{x}\pm s\right)$.

Group	Number of examples	Surgery time (min)	Bleeding volume (mL)	Postoperative hospital stay (d)	Treatment Cost (in ten thousand yuan)
Intervention Group	50	125.60 ± 35.47	8.25 ± 1.77	5.89 ± 1.24	13.25 ± 1.06
Microarray	50	197.83 ± 40.62	208.38 ± 25.94	10.56 ± 2.71	8.34 ± 1.12
*t*		9.471	54.428	11.080	22.514
*P*		<0.001	<0.001	<0.001	<0.001

### 3.2 Comparison of aneurysm occlusion rates between the two groups

The complete occlusion rate in the interventional group was 88.00% (44/50), which was significantly lower than 100.00% (50/50) in the microsurgical group (Fisher's exact probability test, *P* = 0.026, Table 3).

**Table 3:** Comparison of aneurysm occlusion rates between the two groups [*n*(%)].

Group	Number of examples	Complete occlusion rate	Non-complete occlusion rate
Intervention Group	50	44 (88.00)	6 (12.00)
Microarray	50	50 (100.00)	0 (0.00)
Fisher's exact test	*P*	0.026	

### 3.3 Comparison of the incidence of treatment-related saes within 30 days after surgery between the two groups

The total incidence of treatment-related SAEs within 30 days after surgery in the interventional group was 4.00% (2/50), significantly lower than 16.00% (8/50) in the microsurgical group (*χ*² = 4.000, *P* = 0.045, Table 4).

**Table 4:** Comparison of the incidence of treatment-related SAEs within 30 days after surgery between the two groups [*n*(%)].

Group	Number of examples	Epileptic seizure	Ischemic stroke	Hemorrhagic stroke	Intracranial infection	Total incidence rate
Intervention group	50	0 (0.00)	2 (4.00)	0 (0.00)	0 (0.00)	2 (4.00)
Microarray	50	1 (2.00)	5 (10.00)	1 (2.00)	1 (2.00)	8 (16.00)
*χ* ^2^						4.000
*P*						0.045

### 3.4 Comparison of good functional prognosis rate at 6 months postoperatively between the two groups

The good functional prognosis rate at 6 months postoperatively was 92.00% (46/50) in the interventional group and 98.00% (49/50) in the microsurgical group, with no statistically significant difference between the two groups (*χ*² = 1.895, *P* = 0.169, Table 5).

**Table 5:** Comparison of good functional prognosis rate at 6 months postoperatively between the two groups [*n*(%)].

Group	Number of examples	≤2 points	3 points	4 points	5 points	Functional prognosis rate
Intervention Group	50	46 (92.00)	2 (4.00)	1 (2.00)	1 (2.00)	46 (92.00)
Microarray	50	49 (98.00)	1 (2.00)	0 (0.00)	0 (0.00)	49 (98.00)
*χ* ^2^						1.895
*P*						0.169

### 3.5 Comparison of serum levels of miR-27a and miR-143 between the two groups

There were no statistically significant differences in the preoperative serum levels of miR-27a and miR-143 between the two groups (*P* > 0.05). Compared with preoperative levels, the serum levels of miR-27a and miR-143 in both groups increased significantly at 3 days after surgery (*P* < 0.05), and the levels in the interventional group were significantly lower than those in the microsurgical group at the same time point (all *P* < 0.001, Table 6).

**Table 6:** Comparison of serum levels of miR-27a and miR-143 between the two groups $\left(\bar{x}\pm s\right)$.

Group	Number of examples	miR-27a		miR-143	
		Preoperative	Postoperative 3 d	Preoperative	Postoperative 3 d
Intervention Group	50	1.05 ± 0.17	1.39 ± 0.15^a^	1.18 ± 0.20	1.40 ± 0.19^a^
Microarray	50	1.02 ± 0.14	1.53 ± 0.18^a^	1.15 ± 0.23	1.69 ± 0.16^a^
*t*		0.963	4.225	0.696	8.255
*P*		0.338	<0.001	0.488	<0.001

a *P*<0.05 compared with the preoperative level in the same group.

### 3.6 Comparison of 1-year recurrence rate between the two groups

All patients completed the 1-year follow-up. There were 6 cases of recurrence in the interventional group and no recurrence in the microsurgical group. The 1-year recurrence rate in the interventional group was 12.00%, which was significantly higher than 0 in the microsurgical group (Fisher's exact probability test, *P* = 0.026).

### 3.7 Comparison of postoperative cure rates based on the morphology and location of aneurysms in the two groups

Both sets of analyses indicated that aneurysm anatomical characteristics are a key factor determining the differences in treatment outcomes between the two methods. For aneurysms with favorable anatomical features (narrow neck, apex-neck ratio ≥1.5, no involvement of middle cerebral artery branches, and regular shape), there was no significant difference in complete occlusion rates or 1-year recurrence rates between coil embolization alone and microsurgical clipping (*P* > 0.05). However, for aneurysms with complex anatomical features (wide neck, apex-neck ratio < 1.5, involvement of branches, or irregular shape), interventional treatment resulted in significantly lower complete occlusion rates and higher 1-year recurrence rates (*P* < 0.05). Microsurgical clipping demonstrated better occlusion rates and a lower risk of short-term recurrence in such patients. See Table 7.

**Table 7:** Compares the postoperative cure rates of aneurysm morphology and location between the two groups.

Subgroup type	Subgroup	Number of examples	Complete occlusion rate (%) in the intervention group	Reincidence rate (%) in the intervention group after 1 year	Microscopic total occlusion rate (%)	Microscopic group 1-year recurrence rate (%)	*P*
Tumor neck width	Narrow neck (<4 mm)	62	93.33	6.67	100.00	0	0.237
	Wide neck (≥4 mm)	38	78.95	21.05	100.00	0	0.048
Vertex-neck ratio	AR ≥ 1.5	71	91.43	8.57	100.00	0	0.118
	AR < 1.5	29	80.00	20.00	100.00	0	0.047
Branch inclusion	Non-branch inclusion	58	90.32	6.45	100.00	0	0.121
	Branch inclusion	42	84.62	21.05	100.00	0	0.026
Aneurysm complexity	Non-complex aneurysm	73	90.91	9.09	100.00	0	0.135
	Complex aneurysm	27	75.00	33.33	100.00	0	0.019

## 4 Discussion

Surgical decision-making for the specific population of patients with unruptured MCAA and a history of TIA is one of the major challenges in neurosurgery. Most of these patients are complicated with atherosclerotic stenosis or thrombus shedding from the aneurysm, which makes the aneurysm more prone to rupture under hemodynamic stress and increases the risk of therapeutic operations (16, 17). Therefore, clinical treatment requires a more cautious and individualized risk-benefit assessment to ensure that patients obtain maximum benefits.

With the widespread promotion of the minimally invasive concept and the continuous development of interventional techniques, endovascular interventional therapy has become a new trend in the treatment of aneurysms (18, 19). The results of this study showed that the operation time, blood loss and hospital stay in the interventional group were all less than those in the microsurgical group, which was basically consistent with the research results of Zhang et al. (20), indicating that endovascular interventional therapy has the advantages of less blood loss, higher surgical efficiency and faster postoperative recovery. This may be due to the minimally invasive endovascular operation in interventional therapy, which avoids complex surgical procedures such as craniotomy and brain tissue retraction, thus reducing the operation time and intraoperative blood loss. In addition, this study found that the incidence of treatment-related SAEs within 30 days after surgery in the interventional group was 4.00%, lower than 16.00% in the microsurgical group, indicating that endovascular interventional therapy can reduce the risk of treatment-related SAEs. Zhang et al. (20) reported that there was no significant difference in surgical complications between endovascular interventional therapy and microsurgical clipping in the treatment of aneurysmal subarachnoid hemorrhage, which was inconsistent with the results of this study. Kang et al. (21) conducted a systematic analysis of these two techniques in the treatment of unruptured aneurysms, and the results showed that the short-term complications of endovascular interventional therapy were lower than those of microsurgical clipping, with the main difference being the higher incidence of cerebral ischemic events after microsurgical treatment. This study also showed that the incidence of ischemic stroke in the microsurgical group was 10.00%, accounting for the highest proportion in the total incidence of treatment-related SAEs. This may be because the operation of microsurgical clipping is prone to injury to the perforating vessels of the middle cerebral artery and induce vasospasm, thus increasing the risk of postoperative deep cerebral infarction. Especially for patients with a history of TIA, their cerebrovascular reserve function is poor, which may further increase the incidence of ischemic stroke (22–24). However, 4% of patients in the interventional group also developed ischemic stroke, which may be related to the history of TIA in the patients. This result also suggests that a reasonable antiplatelet therapy strategy should be formulated for this population to prevent cerebral infarction, and platelet changes should be closely monitored to avoid increasing the risk of hemorrhagic stroke. At the same time, this study also showed that the treatment cost in the interventional group was higher than that in the microsurgical group, indicating that the treatment cost of endovascular interventional therapy is more expensive.

Although endovascular interventional surgery is a more minimally invasive technique and is favored by clinicians, some scholars believe that microsurgical clipping still has significant advantages in vascular embolization, which can improve prognostic function and reduce the risk of recurrence (25–28). The results of this study showed that the complete occlusion rate in the interventional group was 88.00%, lower than 100.00% in the microsurgical group, and the recurrence rate was 12.00%, higher than 0 in the microsurgical group, indicating that microsurgery can improve the complete occlusion effect of aneurysms and reduce the risk of postoperative recurrence in the treatment of unruptured MCAA with a history of TIA. The reason may be that microsurgical clipping is performed under direct microscopic vision, which can clearly distinguish the aneurysm neck, parent artery and surrounding perforating vessels, thus achieving complete occlusion of the aneurysm (29, 30). In addition, the occurrence of TIA may be related to microthrombus shedding from the aneurysm or the presence of atherosclerotic plaques. Microsurgery is conducive to the direct removal of intraluminal thrombus and evaluation of plaques during the operation, fundamentally addressing the causes of TIA (31, 32). For patients with difficult clipping, immediate decisions can be made on whether to perform vascular reconstruction to maximize the benefits of patients. Previous studies have suggested that the levels of miR-27a and miR-143 can predict the postoperative prognosis of patients with aneurysms, and lower levels indicate a poor prognosis (33, 34). The results of this study showed that the serum levels of miR-27a and miR-143 in the interventional group at 3 days after surgery were lower than those in the microsurgical group, suggesting that patients receiving endovascular interventional therapy have a risk of poor prognosis. miR-27a and miR-143 have a negative regulatory effect on neuroinflammatory responses, and can inhibit neuronal apoptosis and reduce brain injury (35, 36). Preoperative aneurysm compression of surrounding nerves or brain tissue may lead to a decrease in the levels of miR-27a and miR-143. Microsurgical clipping can completely embolize the aneurysm and completely relieve the space-occupying effect, thus reducing the secondary injury of brain tissue or nerves, resulting in a more significant increase in the levels of the above indicators at 3 days after surgery. However, there was no significant difference in the good functional prognosis rate between the two groups in this study, which may be related to the small sample size and short observation time of this study, leading to insufficient statistical power, and further verification is needed in future studies.

In terms of patient selection, contemporary neurosurgical treatment approaches are typically determined based on the patient's age, comorbidities, and the anatomical characteristics of the aneurysm. For patients under 60 years old with no severe comorbidities and a long expected lifespan, microsurgical clipping is generally recommended as it provides more durable aneurysm occlusion, reduces the risk of long-term recurrence, and eliminates the need for prolonged imaging follow-up and additional interventions. In contrast, for patients over 70 years old with multiple underlying diseases who cannot tolerate craniotomy, endovascular treatment is the preferred option due to its lower perioperative risks, faster recovery, and reduced incidence of perioperative complications. Additionally, for aneurysms with narrow necks and small sizes, the difference between the two treatment methods is minimal. However, for wide-necked or complexly shaped aneurysms, microsurgical clipping offers clear advantages, which is consistent with the subgroup analysis results in this study.

However, this study has its limitations. Firstly, it is a single-center, small-sample prospective cohort study rather than a rigorously designed randomized controlled trial, so there is a potential for selection bias. The choice of treatment method was partly based on the patient's preferences and the clinician's assessment, which may affect the study results. Secondly, the follow-up period in this study was only one year. For aneurysm treatment, especially endovascular treatment, recurrence and durability of treatment outcomes are long-term outcomes that require follow-up over 3–5 years or longer to accurately assess. Therefore, the one-year recurrence rate reported in this study does not fully reflect the long-term durability of either treatment method. We cannot draw definitive conclusions about the long-term effects of these treatments based on these short-term findings. Further long-term follow-up is necessary to confirm these results. Additionally, this study used only conventional coiling techniques for endovascular treatment and did not include patients who received modern adjunctive interventional procedures. This limits the generalizability of the study's findings to current practices in modern neurointerventional therapy, and the results cannot be directly applied to treatments involving stent-assisted techniques, balloon angioplasty, or flow-diverting devices.

In conclusion, both microsurgical clipping and endovascular interventional embolization are feasible therapeutic strategies for patients with unruptured MCAA and a history of TIA. Interventional techniques have the advantages of shorter operation time, less blood loss, faster postoperative recovery and higher perioperative safety, which are in line with the minimally invasive concept, while microsurgery achieves a higher complete occlusion rate and lower recurrence risk, and can achieve the goal of radical cure. Therefore, microsurgery provides the most durable solution for young patients with a long life expectancy, who pursue radical cure and can tolerate craniotomy; while interventional techniques are an important minimally invasive alternative for elderly patients with multiple comorbidities, who cannot tolerate craniotomy or have extremely high requirements for perioperative safety. Clinical therapeutic decisions should be made based on individual patient conditions to formulate personalized regimens. However, this study also has limitations: as a single-center, small-sample randomized controlled trial, only the 6-month functional prognosis and short-term recurrence rate are reported, and the extrapolation of the results and long-term prognosis need to be further verified by multi-center, large-sample studies; in addition, this study initially observed the postoperative changes of miR-27a and miR-143, but the exact pathophysiological mechanism of the changes in the levels of these microRNAs and their specific association with the long-term stability of aneurysms and vascular repair processes have not been elucidated, which requires in-depth exploration in subsequent basic research.

## Data Availability

The original contributions presented in the study are included in the article/Supplementary Material, further inquiries can be directed to the corresponding author.
